# The Plastid-Encoded RNA Polymerase-Associated Protein PAP9 Is a Superoxide Dismutase With Unusual Structural Features

**DOI:** 10.3389/fpls.2021.668897

**Published:** 2021-06-30

**Authors:** Adrien Favier, Pierre Gans, Elisabetta Boeri Erba, Luca Signor, Soumiya Sankari Muthukumar, Thomas Pfannschmidt, Robert Blanvillain, David Cobessi

**Affiliations:** ^1^Université Grenoble Alpes, CEA, CNRS, IBS, Grenoble, France; ^2^Université Grenoble-Alpes, CNRS, CEA, INRA, IRIG-LPCV, Grenoble, France

**Keywords:** plastid-encoded RNA polymerase, iron superoxide dismutase, chloroplast biogenesis, NMR, X-ray crystallography

## Abstract

In Angiosperms, the plastid-encoded RNA polymerase (PEP) is a multimeric enzyme, essential for the proper expression of the plastid genome during chloroplast biogenesis. It is especially required for the light initiated expression of photosynthesis genes and the subsequent build-up of the photosynthetic apparatus. The PEP complex is composed of a prokaryotic-type core of four plastid-encoded subunits and 12 nuclear-encoded PEP-associated proteins (PAPs). Among them, there are two iron superoxide dismutases, FSD2/PAP9 and FSD3/PAP4. Superoxide dismutases usually are soluble enzymes not bound into larger protein complexes. To investigate this unusual feature, we characterized PAP9 using molecular genetics, fluorescence microscopy, mass spectrometry, X-ray diffraction, and solution-state NMR. Despite the presence of a predicted nuclear localization signal within the sequence of the predicted chloroplast transit peptide, PAP9 was mainly observed within plastids. Mass spectrometry experiments with the recombinant *Arabidopsis* PAP9 suggested that monomers and dimers of PAP9 could be associated to the PEP complex. In crystals, PAP9 occurred as a dimeric enzyme that displayed a similar fold to that of the FeSODs or manganese SOD (MnSODs). A zinc ion, instead of the expected iron, was found to be penta-coordinated with a trigonal-*bipyramidal* geometry in the catalytic center of the recombinant protein. The metal coordination involves a water molecule and highly conserved residues in FeSODs. Solution-state NMR and DOSY experiments revealed an unfolded C-terminal 34 amino-acid stretch in the stand-alone protein and few internal residues interacting with the rest of the protein. We hypothesize that this C-terminal extension had appeared during evolution as a distinct feature of the FSD2/PAP9 targeting it to the PEP complex. Close vicinity to the transcriptional apparatus may allow for the protection against the strongly oxidizing aerial environment during plant conquering of terrestrial habitats.

## Introduction

In the green lineage, the photosynthetic reactions in the chloroplast convert light energy into chemical energy with the release of di-oxygen. Other metabolic pathways take place in chloroplasts such as the biosynthesis of amino acids, fatty acids, vitamins, and hormones. Hence, the chloroplast functions sustain most life forms on Earth (Jarvis and López-Juez, 2013). According to the endosymbiosis theory of the origin of organelles, chloroplasts have evolved from a single ancient cyanobacterium engulfed around 1.5 billion years ago into a mitochondriate proto-eukaryote (Bobik and Burch-Smith, 2015). During evolution, a massive gene transfer occurred from the cyanobacterium into the nucleus of the host cell (Martin et al., 2002). Thus, the nuclear genome could encode from 1500 to 4500 chloroplast proteins whereas the plastid genome (plastome) encodes for about hundred proteins (Zybailov et al., 2008). The plastome (cpDNA) mainly encodes: (1) components of the plastid gene expression machinery (RNA polymerase, ribosomal proteins, tRNAs, and rRNAs), (2) subunits of each major functional photosynthesis-related complex (e.g., RuBisCO, Photosystem I and II, the cytochrome *b*_6_*f* complex, NADPH dehydrogenase, and ATP synthase), and (3) a few proteins involved in other processes (e.g., ClpP1 and YCF3) (Sugiura, 1992; Majeran et al., 2012; Yu et al., 2014). Hence, the vast majority of chloroplast proteins are encoded by the nuclear genome. The pre-proteins are imported into the chloroplast from the cytosol mainly by the TOC-TIC machinery of the chloroplast envelope that recognizes and cleaves specific transit peptides (cTPs) at their N-terminal extremity (Jarvis, 2008). Once in the stroma, the proteins are properly folded. Since most of the protein complexes in the chloroplast contain nuclear and chloroplast-encoded proteins, coordination in expression of both genomes is essential (Liebers et al., 2017).

Two RNA polymerases are involved in plastid transcription: a nuclear-encoded RNA polymerase (NEP) and the plastid-encoded RNA polymerase (PEP). The NEP, a T3–T7 bacteriophage type RNA polymerase, transcribes the *rpo* genes (rpoA, B, C1, and C2), encoding the four subunits of the catalytic core of the PEP, and other housekeeping genes (Kremnev and Strand, 2014; Börner et al., 2015). During chloroplast biogenesis, the PEP core is reshaped in a multi-subunit RNA-polymerase of at least 16 different proteins (MW: ∼1 MDa), which mainly transcribes photosynthesis related genes. The active PEP complex is composed of four rpo core subunits, and 12 nuclear-encoded PEP-associated proteins (PAPs) (Pfannschmidt et al., 2015). Mutations in most of the *pap* genes yield albino/ivory plants incapable of photosynthesis with a defect in the expression of PEP-dependent genes indicating that the PEP is not fully functional (Pfannschmidt et al., 2015). This shared phenotype triggered the idea of a PAPs-related developmental block corresponding to an epistasis effect. This effect occurs when all components are required for the stability of the entire complex ensuring that photosynthesis could be launched only if all the functions are present (Liebers et al., 2018).

The PAPs can be divided into four groups according to their hypothetical functions (Yu et al., 2014). PAP sequence analyses and biochemical studies allowed to characterize four PAPs with potential known catalytic activities: PAP4, PAP7, PAP9, and PAP10. PAP7 belongs to methyltransferases (Gao et al., 2011), PAP10 is a thioredoxin (TrxZ) (Steiner et al., 2011) while PAP4 (FSD3) and PAP9 (FSD2) are both iron superoxide dismutases (FeSOD) (Myouga et al., 2008). Formation of superoxide radicals mainly occurs in electron transport chains of photosynthesis and respiration. Therefore, PAP4 and PAP9 may serve as protection against oxidative stresses generated during the first activities of the photosynthetic apparatus (Pfannschmidt et al., 2015). Indeed, superoxide radicals can damage sulfur containing amino acids, metals, and Fe-S clusters. SODs are cellular defenses against superoxide by catalyzing the dismutation of superoxide into hydrogen peroxide according to the overall reaction: 2O_2_^–^ + 2H^+^ → H_2_O_2_ + O_2_ (Pfannschmidt, 2003; Abreu and Cabelli, 2010).

Besides the MnSODs and the copper-zinc SODs (Cu/ZnSODs, where Cu is the redox center), three iron superoxide dismutases (FeSODs) were characterized in plants. Dimeric MnSODs are found in the matrix of the mitochondria, with one Mn ion per monomer. Cu/ZnSODs are dimeric SODs found in the cytosol, peroxisomes, and plastids. Each monomer contains one Cu and one Zn ion. FeSODs are dimeric enzymes with one iron ion bound to each monomer. The fold of the FeSOD monomer is roughly similar to that of the MnSOD monomer and is completely different from the Cu/ZnSODs (Pilon et al., 2011). In plants, FSD1 is a cytoplasmic FeSOD, while PAP4 and PAP9 are FeSODs only observed in the chloroplast, both associated to the PEP (Myouga et al., 2008; Steiner et al., 2011). Surprisingly, the oligomeric assembly of PAP4 and PAP9 differ from that observed for FeSODs. PAP9 was reported as being a monomer in the PEP and PAP4 as a trimer (Steiner et al., 2011). In *Arabidopsis thaliana*, PAP4 and PAP9 could form a heterodimeric complex in the chloroplast nucleoids (Myouga et al., 2008). The *pap4–pap9* double mutant displayed an albino phenotype with no chloroplast development while the *pap4* or *pap9* single inactivation mutants showed pale green phenotypes and sensitivity to oxidative stress indicating some compensation effect but no full redundancy between the two proteins (Myouga et al., 2008). These observations strongly suggested that a heterodimeric complex PAP4/PAP9 could protect the transcriptionally active chromosome (TAC) during the early stages of chloroplast development from the superoxide radical produced during photosynthesis in the thylakoid membranes (Myouga et al., 2008). To better characterize PAP9 and understand how plastid-localized FeSODs were embedded in the PEP, we studied PAP9 using phylogenetic approaches, *in planta* experiments, mass spectrometry, X-ray diffraction, and solution-state NMR.

## Materials and Methods

### Accessions

PAP9 At5g51100; accessions from the green lineage are given in Supplementary Table 1. Full-length coding sequences were retrieved from Blastp (Supplementary Table 2). The protein sequences were aligned using Clustal Omega^1^. The prediction of chloroplast pre-sequences (Supplementary Table 3) were established using ChloroP^2^ (Emanuelsson et al., 1999). The predictions of the nuclear localization signals (NLS) were performed using NLS_Mapper^3^ and are given in Supplementary Table 4 (Kosugi et al., 2009). Clustal Omega color-code as followed: [red (AVFPMILW): small + hydrophobic (includes aromatic Y); blue: (DE), acidic; magenta: (RHK), basic; green: (HSTYHCNGQ), hydroxyl + sulfhydryl + amine + G].

### Peptide Synthesis

The peptide ^226^QREQEGTETEDEENPDDEVPEVYLDSDIDVSE VD^259^ corresponding to the last 34 residues of PAP9 was synthesized by Proteomic Solution with a purity (HPLC) of 98.29%. Its molecular mass (MW: 3925.85 Da) was checked using mass spectrometry.

### Transient Transformation of Onion Cells

Gold Carrier Particles (Seashell technology) were coated with 1 μg of the expression vector and 1 μg of an internal control such as PAP10-RFP (Liebers et al., 2020). Gold particles were delivered into onion cells using a particle gun (BioRad). The transformed cells were allowed to express the construct for 16–24 h before fluorescence observation using proper filters. Signal profiles of the two fluorescence channels were acquired on pictures using ImageJ.

### Cloning and Vector Construction

PAP9^Δ*cTP*^ (271 aa/31 kDa) in pBB408 corresponds to PAP9^Δ*cTP*^-6His in the pEt21d backbone: RT-PCR fragment was obtained from seedling cDNA amplified with oP9ΔcTP_FNco (5′-CCATGGGTGTTATCACAGCTGG)/oP9_RNot (5′-GCGGCC GCGTCAACCTCAGATACATCGATG), A-tailed and cloned in pGem-Teasy (pBB399a) then digested with *Nco*I, *Not*I and cloned in pET21d. PAP9-GFP in pAF04 (pEZS-NL backbone, Stanford): RT-PCR fragment was obtained from seedling cDNA amplified with oPAP9_FXho (5′-CTC GAGATGATGAATGTTGCAGTGACAGCC) and oPAP9_ RBH (5′-GGATCCCCGTCAACCTCAGATACATCGATGTCAC) cloned as above then digested with *Xho*I *Bam*HI and ligated in pEZS-NL. pBB301 (PA10-RFP) was used as internal control (Liebers et al., 2020).

### Protein Expression and Purification

PAP9-6His (for ΔcTP-PAP9-6His) was overexpressed in *E. coli* Rosetta2 strain in LB with 100 μg/mL ampicillin and 34 μg/mL chloramphenicol. 6His-PAP9 (for ΔcTP-6His-PAP9) was overexpressed in *E. coli* Rosetta2 strain in LB with 100 μg/mL ampicillin and 50 μg/mL kanamycin. Cells were grown overnight in 50 mL of LB with antibiotics at 37°C. One liter of LB (with antibiotics) was then inoculated with the first culture to reach an initial OD_600_ of 0.1. Growth was continued at 37°C. When the OD_600_ reached 0.6, the temperature was decreased to 16°C and isopropyl β-D-1-thiogalactopyranoside was added to give a final concentration of 0.5 mM. After an overnight induction, bacteria were harvested at 6619 g, for 25 min, at 4°C. The cell pellet was resuspended in 30 mL of lysis buffer (50 mM Tris–HCl pH 8.0, 0.5 M NaCl, 20 mM imidazole) containing a Complete Protease inhibitor Cocktail tablet (Roche). The lysate was centrifuged at 15,000 *g*, for 40 min, at 4°C. The purification was performed at room temperature. The supernatant was applied onto a NiNTA column in 50 mM Tris–HCl pH 8.0, 0.5 M NaCl, 20 mM imidazole. Proteins were eluted in one step in a buffer containing 50 mM Tris–HCl pH 8.0, 0.1 M NaCl, 300 mM imidazole. Then the eluate was diluted 2 times in 50 mM Tris–HCl pH 8.0 and loaded on a MonoQ column. Elution was performed using a linear NaCl gradient from 0 to 1 M in 50 mM Tris–HCl pH 8.0. The fractions containing PAP9-6His or 6His-PAP9 were pooled and concentrated with an Amicon Ultra 4 mL centrifugal filter and a 10 kDa membrane cut-off before loading on a HiLoad 16/60 Superdex 200 and then eluted with 10 mM Tris–HCl pH 8.0, 50 mM NaCl. The fractions containing the pure protein were pooled and concentrated for further experiments or stored at −20°C with 50% (v/v) glycerol.

^15^N,^13^C-6His-PAP9 was expressed in minimum media M9 supplemented with ^15^NH_4_Cl, ^13^C-glucose and antibiotics. Briefly, 5 mL of LB were inoculated with *E. coli* Rosetta2 stock glycerol overexpressing 6His-PAP9. After 10 h of growing, 1 mL was added to 100 mL of minimum media supplemented as described above. After 1 night growing, when OD_600_ was close to 2, the overnight culture was centrifuged to inoculate 1 L of minimum media M9 supplemented with ^15^NH_4_Cl and ^13^C-glucose and antibiotics. Cell growth, overexpression and purification followed the procedure described above for 6His-PAP9 and PAP9-6His.

### Enzymatic Assays

The superoxide dismutase activity of PAP9 was tested using pyrogallol. The pyrogallol auto-oxidation is characterized by increase of absorbance at 420 nm and superoxide dismutase inhibits the pyrogallol auto-oxidation. Briefly, 7 mM pyrogallol was dissolved in a *Tris*-succinate-EDTA buffer pH 8.2 and the pyrogallol auto-oxidation was followed by monitoring the absorbance increase at 420 nm. After 180 s, PAP9 at several concentrations (50, 100, 200, 500 μM, and 1 mM) or 5 μM Mn-SOD were added into the medium and the absorbance was monitored for further 3 min. Experiments were repeated three times for each concentration and the curves were plotted. Each curve correspond to the average of three enzymatic assays (Supplementary Figure 1).

### LC/ESI and Native Mass Spectrometry

Liquid chromatography electrospray ionization mass spectrometry (LC/ESI-MS) was used to assess the masses of the intact PAP9-6His, and ^15^N,^13^C-6His-PAP9. All solvents were HPLC grade (Chromasolv, Sigma-Aldrich) and trifluoroacetic acid (TFA) was from Acros Organics (puriss, p.a.). Solvent A was 0.03% TFA in water, solvent B contained 95% acetonitrile, 5% water, and 0.03% TFA. A 6210 LC/ESI-TOF mass spectrometer interfaced with an HPLC binary pump system (Agilent Technologies) was used. The mass spectrometer was calibrated in the mass-to-charge (*m/z*) range 300–3000 using a standard calibrant (ESI-L, low concentration tuning mix, Agilent Technologies) before the measurements of protein samples. MS acquisition was carried out in positive ion mode and mass spectra were recorded in the 300–3200 *m/z* range. ESI source temperature was set at 573 K, nitrogen was used as drying gas (7 L/min) and as nebulizer gas (10 psi). The capillary needle voltage was set at 4000 V. Spectra acquisition rate was of 1.03 spectra/s. The MS spectra were acquired and the data processed with MassHunter workstation software (v. B.02.00, Agilent Technologies) and with GPMAW software (v. 7.00b2, Lighthouse Data, Denmark). Immediately before the MS analysis, the protein samples were diluted to a final concentration of 8 μM using solvent A. Samples were kept at 10°C in the autosampler and 8 μL of each sample were injected into the system. They were first trapped and desalted on a reverse phase-C8 cartridge (Zorbax 300SB-C8, 5 μm, 300 μm ID × 5 mm, Agilent Technologies) for 3 min at a flow rate of 50 μL/min with 100% solvent A and then eluted and separated on a RP-HPLC column (Jupiter Proteo, 4 μm, 90 Å, 1 mm ID × 50 mm, Phenomenex) using a linear gradient from 5 to 95% solvent B in 15 min.

PAP9-6His was also analyzed by native MS (Boeri Erba and Petosa, 2015; Boeri Erba et al., 2020). Protein ions were generated using a nanoflow ESI (nano-ESI) source. Nanoflow platinum-coated borosilicate ESI capillaries were bought from Thermo Electron SAS (Courtaboeuf, France). MS analyses were carried out on a quadrupole time-of-flight mass spectrometer (Q-TOF Ultima, Waters Corporation, Manchester, United Kingdom). The instrument was modified for the detection of high masses (Sobott et al., 2002; van den Heuvel et al., 2006). The following instrumental parameters were used: capillary voltage = 1.2–1.3 kV, cone potential = 40 V, RF lens-1 potential = 40 V, RF lens-2 potential = 1 V, aperture-1 potential = 0 V, collision energy = 30–140 V, and microchannel plate (MCP) = 1900 V. All mass spectra were calibrated externally using a solution of cesium iodide (6 mg/mL in 50% isopropanol) and were processed with the Masslynx 4.0 software (Waters Corporation, Manchester, United Kingdom) and with Massign software package (Morgner and Robinson, 2012).

### Solution-State NMR

One milligram of the 34 amino-acids C-terminal peptide of PAP9 was dissolved in 25 mM Na phosphate, pH 6.5 to a final concentration of 1 mM. For assignment of the peptide, homonuclear TOCSY, NOESY, and sensitivity-enhanced ^13^C-HSQC experiments were recorded at 25°C on a Bruker ADVANCE III spectrometer operating at ^1^H frequency of 600 MHz and equipped with a triple resonance pulsed field gradient cryoprobe.

For assignment of 6His-PAP9, 100 μM of ^15^N,^13^C-6His-PAP9 in a 90:10 H_2_O:D_2_O, 10 mM Tris pH 8.0, 50 mM NaCl were used. Heteronuclear 3D Best-TROSY-HNCA, Best-TROSY-HNCACB, Best-TROSY-HNCOCANH (Favier and Brutscher, 2011; Solyom et al., 2013), sensitivity-enhanced ^13^C-HSQC and ^15^N-SOFAST experiments were recorded at 298 K on Bruker ADVANCE III HD spectrometers operating either at ^1^H frequency of 600 or 700 MHz and equipped with a triple resonance pulsed field gradient cryoprobe. [^15^N,^1^H]-TRACT (to estimate the global correlation time) (Lee et al., 2006) and DOSY experiments (for measuring the translational diffusion) (Morris and Johnson, 1992) were recorded at 298 K on a Bruker ADVANCE III HD spectrometer operating at ^1^H frequency of 700 MHz.

### Crystallization, Data Collection, and Structure Resolution

6His-PAP9 and PAP9-6His at 5 mg/mL in 10 mM Tris–HCl, pH 8.0, 50 mM NaCl (+10% glycerol for 6His-PAP9) were subjected to crystallization using the sitting-drop vapor-diffusion technique and the high throughput crystallization facility at the EMBL, Grenoble, at 4°C. Crystallization hits were optimized using Limbro plates, at 293 K. Crystals of PAP9-6His were grown in PEG3350 from 15 to 19%, 0.1 M Bis–Tris pH 6.5, 0.2 M NaNO_3_, for data collection. Crystals of 6His-PAP9 were grown in Bis–Tris pH 7.5, PEG3350 18%, 0.2 M NaNO_3_.

Diffraction data for PAP9-6His were collected on ID23-1 at the European Synchrotron Radiation Facility (ESRF), Grenoble, France, at 100 K, using a PILATUS detector and two crystals. Anomalous data at the peak and after the peak of the zinc K-edge for PAP9-6His and native data for 6His-PAP9 were collected on FIP-BM30A (Roth et al., 2002) at the ESRF, at 100 K, using an ADSC 315r detector. Diffraction data (Table 1) were processed and scaled using XDS (Kabsch, 2010).

**TABLE 1:** Statistics of data collection.

	PAP9-6His	PAP9-6His	PAP9-6His
Wavelength (Å) and beamline	0.976250 (ID23-1)	1.280867 (FIP-BM30A)	1.284809 (FIP-BM30A)
Resolution range (Å)	48.20–2.25 (2.31–2.25)	107.0–2.59 (2.75–2.59)	48.69–3.14 (3.33–3.14)
Space group	C2	C2	C2
Unit cell parameters (Å, °)	a = 214.09, b = 83.01, c = 118.24, β = 115.759	a = 215.36, b = 83.39, c = 118.65, β = 115.57	a = 217.63, b = 83.86, c = 120.33, β = 116.13
Molecules in au	5	5	5
Number of total reflections	321,204 (13,098)	436,955 (66,107)	251,369 (38,638)
Unique reflections	83,998 (5642)	115,026 (17,963)	65,945 (10,382)
Average multiplicity	3.82 (2.32)	3.80 (3.68)	3.81 (3.72)
Data completeness (%)	94.5 (86.0)	99.0 (95.9)	99.2 (96.9)
*R*_sym_ (%)	10.8 (77.9)	13.5 (80.4)	15.1 (69.5)
<I/σ_(I)_>	7.87 (1.03)	8.65 (1.82)	8.87 (2.00)
CC (1/2) (%)	99.5 (60.5)	99.2 (73.0)	99.1 (71.0)

*R_sym_ = *ΣΣ| *I*_*i*_ − *I*_*m*_| /ΣΣ*I*_*i*_, *where I_i_ is the intensity of the measured reflection and I_m_ is the mean intensity of this reflection. Values indicated in parentheses correspond to the statistics in the highest resolution shell.*

Phasing was performed by molecular replacement using Phaser (McCoy et al., 2007) from CCP4 (Collaborative Computational Project, Number 4 (CCP4), 1994). To calculate the phases, the crystal structure of the eukaryotic FeSOD from *Vigna unguiculata* (PDB entry: 1UNF) (Muñoz et al., 2005) was used as a model after modifications based on sequence alignment with PAP9 from *A. thaliana* using CHAINSAW (Stein, 2008) from CCP4. The refinements and rebuilding were done using PHENIX (Adams et al., 2010) and COOT (Emsley et al., 2010), respectively. The model refinements were performed with the non-crystallographic symmetry and the water molecules were added using PHENIX in the last stages of the refinement. Refinement statistics are summarized in Table 2. Atomic coordinates and X-ray data for PAP9-6His were deposited in the PDB with the accession number 7BJK. Since 6His-PAP9 is similar to PAP9-6His, the diffraction data and the 3D-structure were not reported in the PDB.

**TABLE 2:** Refinement statistics.

	PAP9-6His
Resolution (Å)	48.20–2.25 (2.28–2.25)
*R*_cryst_ (σ_*F*_ = 0) (%)	17.94 (33.96)
*R*_free_ (σ_*F*_ = 0) (%)	22.10 (38.11)
Number of atoms	8997
Water molecules	399
B average (Å^2^)	51.82
RMSD bonds (Å)	0.007
RMSD angle (°)	0.884
Ramachandran favored (%)	91.5
Ramachandran allowed (%)	7.4
Ramachandran disallowed (%)	0.5

*Values indicated in parentheses correspond to the statistics in the highest resolution shell. R_cryst_* = Σ| | *F*_*obs*_| − | *F*_*calc*_| | /Σ| *F*_*obs*_|. *R*_*free*_*(Brünger, 1992) is the same as R_cryst_ but calculated for 5% data omitted from the refinement.*

## Results

### Phylogeny of PAP9 in the Green Lineage

**FIGURE 1:**
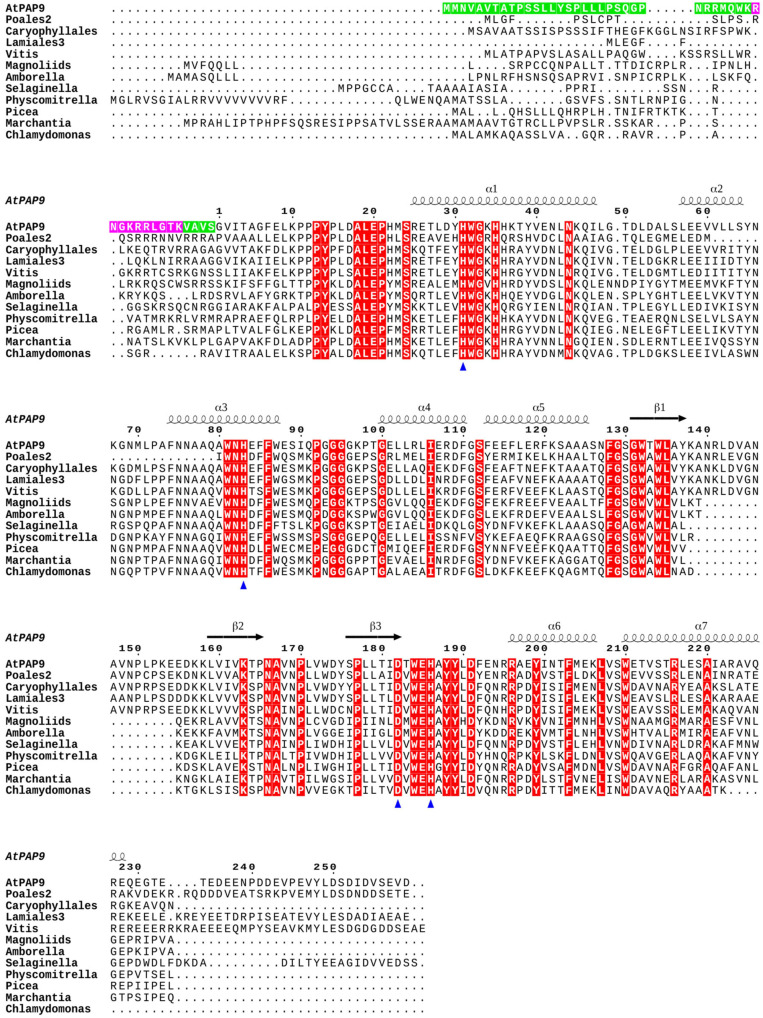
PAP9 secondary structures mapping on a sequence alignment including orthologous proteins from different clades of the green lineage. The PAP9 secondary structure from *Arabidopsis thaliana* is drawn as followed: the α-helices are displayed as squiggles and β-strands as arrows. The conserved residues are highlighted in red. The residues involved in the metal binding, Zn^2+^ in the crystal structure of *A. thaliana* PAP9, are indicated with a blue triangle. The cTP and NLS of the *A. thaliana* PAP9 are highlighted in green and magenta, respectively. The drawing was prepared using ESPript (Robert and Gouët, 2014).

**FIGURE 2:**
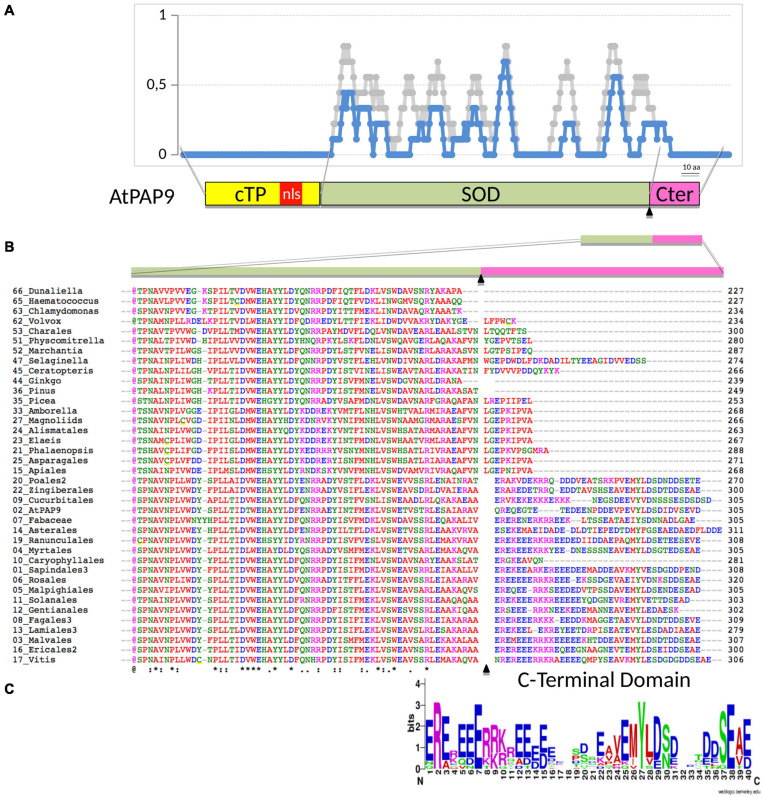
Sequence alignment of predicted orthologous PAP9 protein found in representatives of phylogenetic clades as described in Supplementary Table 1. **(A)** Amino acid identity (in blue) and similarity (in light gray) given in a 10-aa moving ratio. The alignment corresponds to the full dataset given in Supplementary Table 2 marked with a cross (column named in this figure). Schematic illustration of the PAP9 domains: cTP, chloroplast transit peptide in yellow; NLS, predicted nuclear localization signal in red; SOD, superoxide dismutase domain in khaki; C-terminal domain in magenta. **(B)** Amino acid partial alignment of the accessions as above, the 2-digit number corresponds to the clade given in Supplementary Table 1. cTP, transit peptide as predicted with ChloroP1.1 (www.cbs.dtu.dk) underlined in yellow and described in Supplementary Table 3. (*), (:), or (.), conserved, strongly similar or weakly similar amino acid properties (standards from www.uniprot.org). Amino acids colors as in Clustal Omega [red (AVFPMILW): small + hydrophobic (includes aromatic Y); blue (DE): acidic; magenta (RHK): basic; green (STYHCNGQ): hydroxyl + sulfhydryl + amine + G). bNLS, bipartite NLS as predicted with NLS mapper (http://nls-mapper.iab.keio.ac.jp). **(C)** AA sequence logo (Crooks et al., 2004) generated on the C-terminal domain alignment of PAP9 with the 40-aa-long stretch from clade 22 (Zingiberales) to clade 17 (Vitis) excluding clade 10 (Caryophyllales).

Significant sequence similarities with At-PAP9 were found as early as in clades representing the chlorophytes, indicating that salt-water algae acquired plastid-localized SODs early in evolution. However, sequence alignments (Figure 1) identified a critical domain, outside of the SOD catalytic domain (Figure 2A), at the C-terminal (C-ter) of the protein, which had strongly changed during evolution. Whereas absent in early separated clades (as represented by Chlamydomonas), a significant insertion after the last well-conserved arginine (Arg262) is found in *Selaginella* with a large proportion of acidic residues representing one third of the amino acids (Figure 2B). The C-terminal of PAP9 in its long form (i.e., 40 residues) is not essential in higher Angiosperms since different clades have a shorter domain of approximately 20 residues in Physcomitrella, basal clades of the ANA grade, Apiales from Eudicots, Alismatales, and Asparagales from Monocots. Interestingly, the PAP9 C-terminus is either totally absent in Gyngko and Pinus or present as the short sequence in Picea, suggesting that there is no *bona fide* PAP9 referring to the involvement of the protein to the PEP function. These observations corroborate the hypothesis according to which Gymnosperms had favored a different use of PEP complex canceling the use of some PAPs that are not found anymore in the clade. In most Eudicots, a largely acidic tail with a well-conserved tyrosine (Figure 2C) may be involved in the PEP function as it could also play the role of electron donor with manganese clusters or as a signaling residue.

### Subcellular Localization of PAP9-GFP Proteins

**FIGURE 3:**
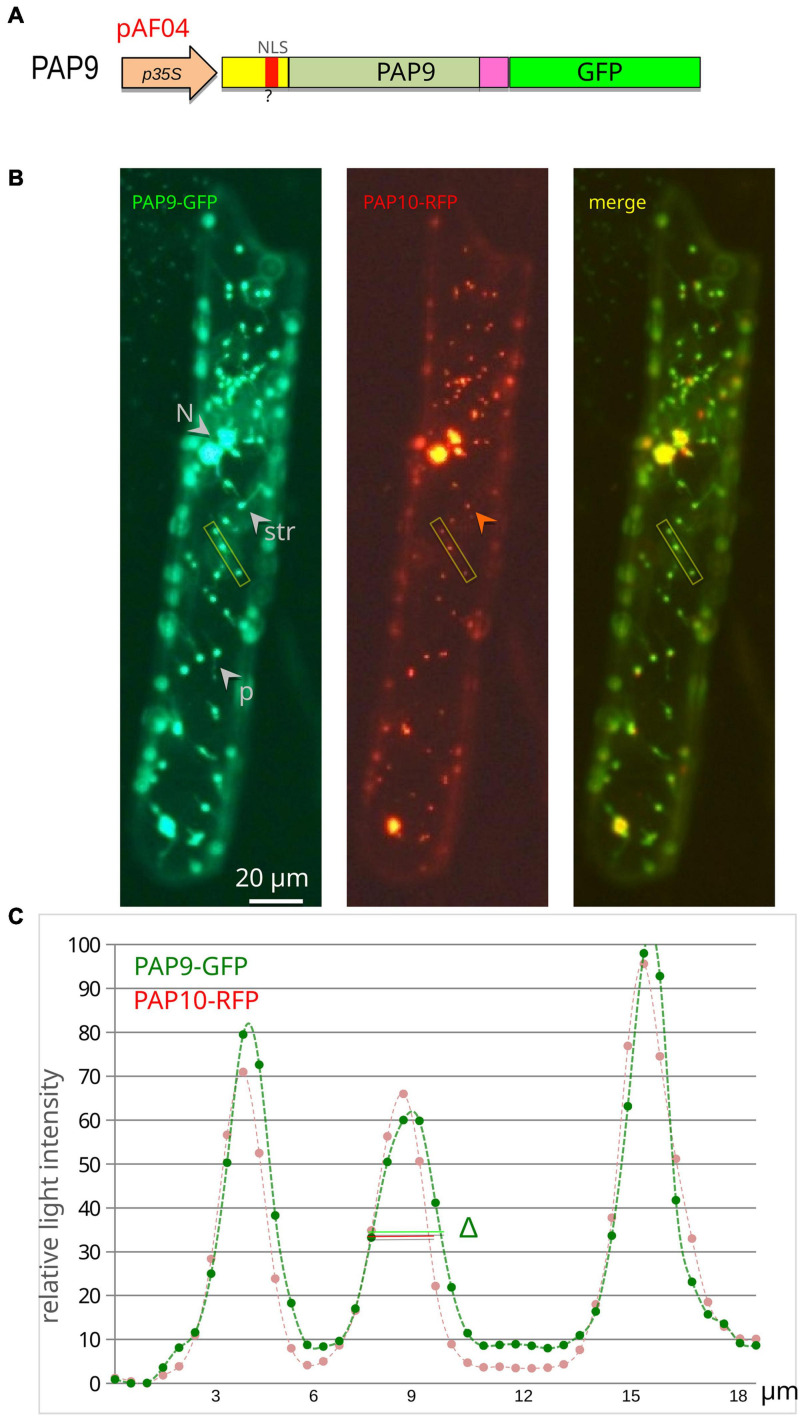
PAP9 is localized in plastids. **(A)** Schematic illustration of the PAP9-GFP construction in pAF04. p35S, CaMV35S promoter region; cTP, chloroplast transit peptide in yellow; ?, predicted NLS (nuclear localization signal) in red; C-terminal domain in magenta; GFP in green. **(B)** Transiently expressed PAP9-GFP in onion epidermal cells. N, nucleus; str, stromule; p, plastid. The red arrowhead points to the absence of red fluorescence in stromules. The yellow rectangle represents the analyzed segment in panel **(C)**. **(C)** Fluorescent signal quantitative profile on an 18-μm-long segment of the image across three plastids. Δ represents the difference in width of the GFP signal compared to the red signal of PAP10-RFP.

Some of the proteins associated to the PEP, like PAP9, possess a predicted NLS (Pfannschmidt et al., 2015). However, the putative NLS of PAP9 (Figure 2A and Supplementary Table 4) is nested within the cTP (Figure 2A and Supplementary Tables 3,4), which is conceptually cleaved off during plastid import through the TOC/TIC machinery. Hence the question arises whether the predicted sequence is actually a *bona fide* NLS. Since the NLS sequence at this position is not conserved in other species, it does not likely play an important role in PAP9 localization. This is experimentally supported by the transient localization of PAP9-GFP (Figures 3A,B), which appears to be mostly plastidial. However, the clear labeling of the stromules (Figure 3B), indicates that a part of the pool of fluorescent molecules is found in the stroma, released from the PEP/PAP complex. In some images, we could also detect some signals in the cytosol and nucleus (Supplementary Figure 2). The GFP fluorescent profile across plastids is more spread than that of the RFP, indicating that the PAP9-GFP signal is not as restricted as that of PAP10-RFP used here as specific marker of the PEP complex (Liebers et al., 2020). The translational fusion of GFP at the C-terminus may alter the function of the corresponding domain so that the localization may not reflect precisely that of PAP9. Such a perturbation has been observed for HMR/PAP5 (Chen et al., 2010) and pTAC6/PAP8 (Liebers et al., 2020) for which C-terminal GFP fusions alter the localization and/or the functionality of the protein.

### Mass Spectrometry Analyzes

**FIGURE 4:**
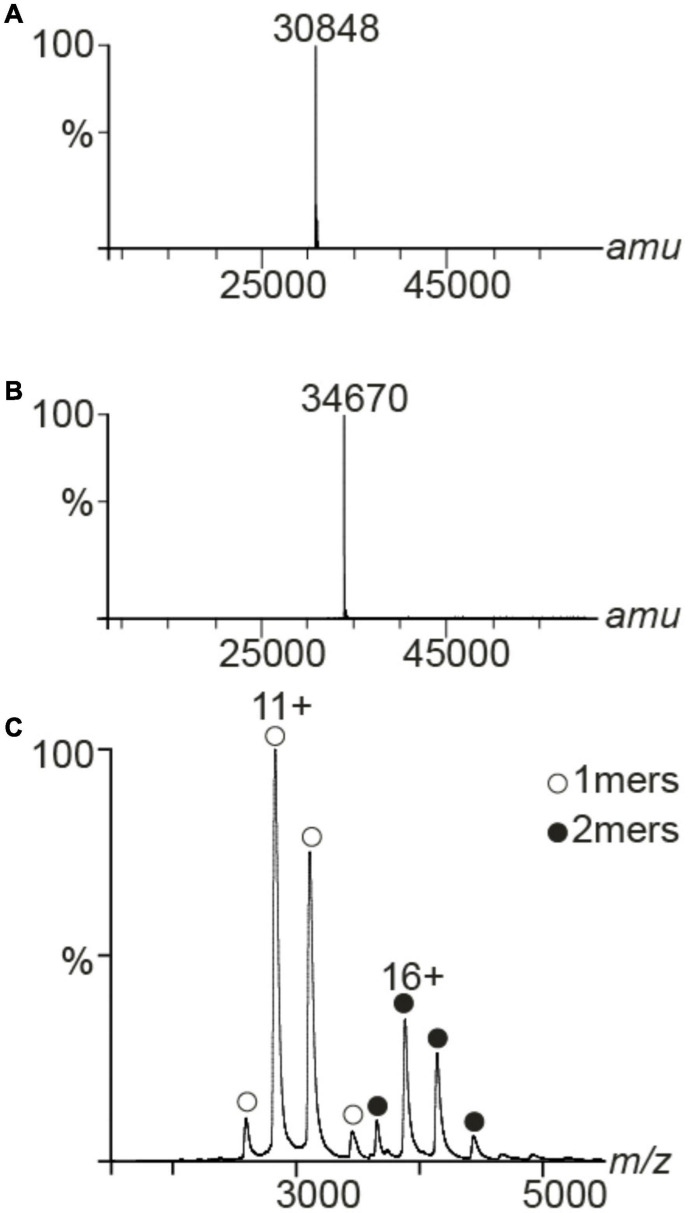
MS spectra of PAP9. Deconvoluted spectra of PAP9-6His **(A)** and ^15^N,^13^C-6His-PAP9 **(B)**. Under denaturing conditions the accurate mass of PAP9-6His was 30,848 and 34,670 Da for ^15^N,^13^C-6His-PAP9. **(C)** Native MS spectrum of the PAP9-6His. It formed two distinct oligomers, such as monomers (1mer, 30,848 ± 1 Da) and dimers (2mers, 61,697 ± 2 Da).

We utilized MS to assess the mass of PAP9-6His and ^15^N,^13^C-6His-PAP9 under denaturing conditions. The experimental mass of PAP9-6His was 30,848 Da, matching the amino acidic sequence 1-270 (Figure 4A) and ^15^N,^13^C-6His-PAP9 displayed a mass of 34,670 Da. The calculated mass of the fully labeled protein is 34,801 Da, taking into account Met at N-terminal that has not been cleaved because the second residue before the 6His-Tag is Lys (Hirel et al., 1989); the difference between both mass resulting from an incomplete labeling (Figure 4B). To investigate the oligomeric state of PAP9-6His, we used native MS and we detected monomers and dimers (Figure 4C).

### X-Ray Structure Analyzes

**FIGURE 5:**
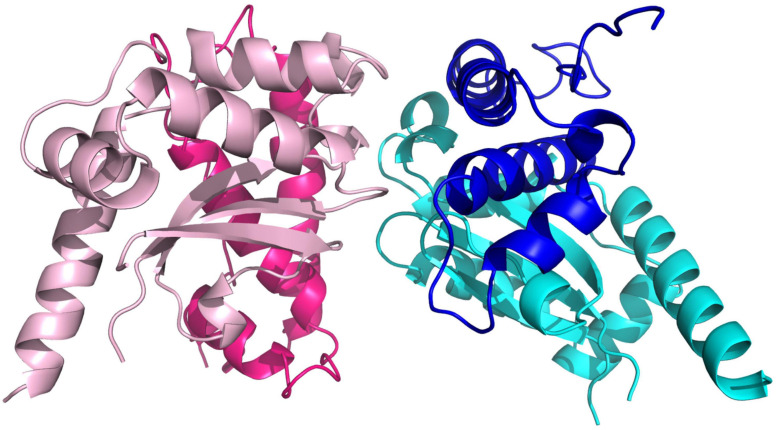
View of the PAP9 dimer. The β-strands are drawn in arrows and the α-helices are represented in ribbons. The N-terminal domains are colored in dark pink and dark blue. The C-terminal domains are in cyan and light pink.

**FIGURE 6:**
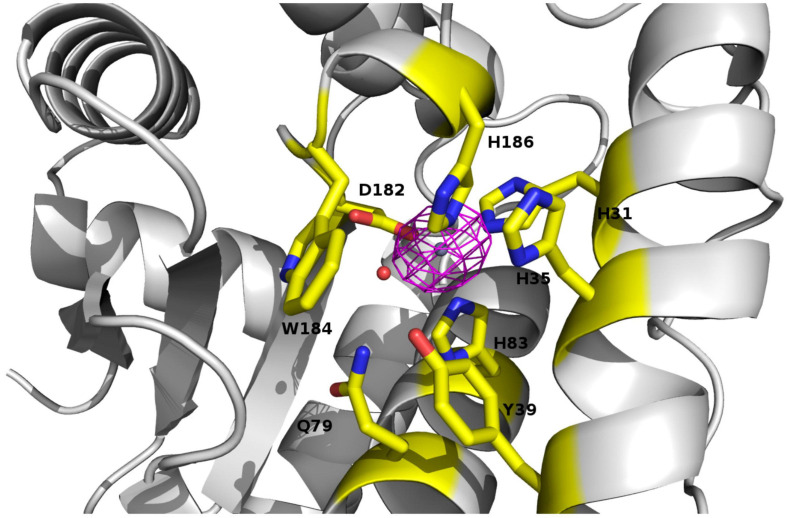
View of the catalytic site of PAP9 superimposed with the anomalous electron density map calculated at the zinc K-edge. Residues of the catalytic site and closing the catalytic site are drawn in sticks. The zinc ion is drawn as gray sphere. The water molecule corresponding to the hydroxide ion is represented as a red sphere.

Five molecules of PAP9 are in the asymmetric unit. Four of them form two dimers. The fifth interacts with a molecule from another asymmetric unit to form also a dimer. Both monomers in the dimer are related by a non-crystallographic twofold axis. The monomers are very similar with a value of root mean square deviation (RMSD) ranging from 0.14 to 0.21 Å between monomers when calculated between the Cα atoms. The buried area calculated using PISA (Krissinel and Henrick, 2007) in the dimer interface is 1785 Å^2^. PAP9 is folded in two domains similar to those observed in FeSODs or MnSODs. The N-terminal domain extends from Gly1 to Gly93 and contains three α-helices. The C-terminal domain (Gly94–Gln229) displays an α/β fold with a three anti-parallel β-strands sandwiched by four α-helices and the N-terminal domain (Figure 5). No electron density is observed for residues from Arg141 to Glu155 and for the last 29 residues from Gly231 to Asp259 suggesting flexibility. Crystallographic analysis of the 6His-PAP9, produced to decrease the C-terminal flexibility, did not allow to better observe the electron density of the C-terminal part and the structures of 6His-PAP9 and PAP9-6His were similar. The catalytic center is at the interface of the N- and C-terminal domains. Surprisingly a zinc ion, instead of the expected iron ion, is penta-coordinated in the catalytic center. Anomalous difference electron density map calculated at the zinc K-edge showed a strong peak of anomalous density (Figure 6) while the map computed with diffraction data collected just after the zinc K-edge does not show any strong peak. The zinc ion is penta-coordinated by the His31, His83 side chains of the N-terminal domain, the Asp182, and His186 side chains from the α/β fold domain, and a water molecule supposed to mimic the position of the hydroxide ion (Figure 6). The arrangement of the five coordinating ligands forms a trigonal bipyramid with His31 and the water molecule as the axial ligands. The side chains of His35, Tyr39, Gln79, and Trp184 close the catalytic site (Figure 6). Since PAP9 mainly binds Zn^2+^ in our expression/purification steps no catalytic activity could be observed excepted at very high PAP9 concentrations (Supplementary Figure 1).

### Structure Comparisons and the PAP9 Family

**FIGURE 7:**
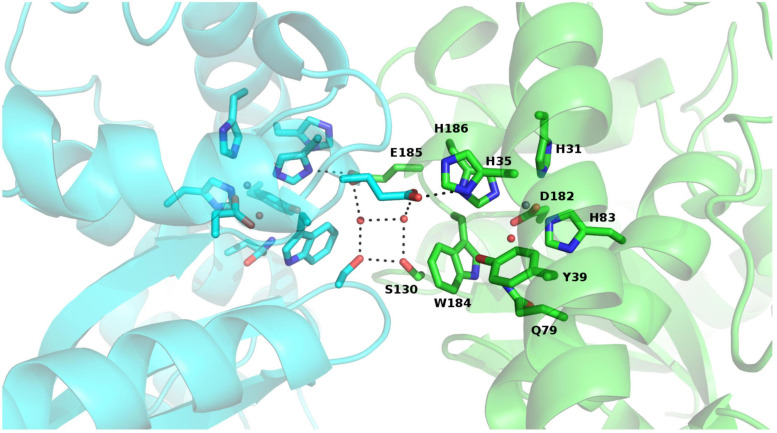
View of the conserved interactions between both monomers of PAP9 *and* observed in FeSODs, with *each monomer* of PAP9 in a different color. The residues involved are drawn in sticks, the hydrogen bonds are represented in dark dashed lines and the water molecules are shown as spheres. The zinc ion is drawn as gray sphere. The β-strands are drawn in arrows and the α-helices are represented in ribbons.

Rms deviations calculated using PDBefold (Krissinel and Henrick, 2004) between the monomer of PAP9 and more than 200 monomers of SODs from the PDB range from 0.71 Å (PAP9 vs. FeSOD from *V. unguiculata*, PDB entry: 1UNF) to 1.6 Å with the FeSOD from *Aquifex pyrophilus* (PDB entry: 1COJ) (Lim et al., 1997). The structure comparisons revealed that the fold of PAP9, the ligands involved in the metal coordination and residues closing the catalytic site are conserved. Dimer interface comparison with FeSOD from *V. unguiculata* revealed also a conservation of residues involved in interactions by hydrogen bonds between the subunits. The Glu185 carboxylate group from one monomer interacts with the Ser130 hydroxyl group involving a water molecule and also with the His186 imidazole ring of the catalytic center from the other monomer. Additionally, the hydroxyl group of Ser130 interacts with the hydroxyl group of Ser130 from the other monomer (Figure 7). The main difference originates from the metal center occupied by a zinc ion in AtPAP9 instead of an iron ion. The conserved interaction described in FeSOD from *V. unguiculata* between His35 of one monomer and Tyr188 of the other monomer is not observed in PAP9. The residues Gly156 to Ser164 of the cytosolic FeSOD from *V. unguiculata* corresponding to Val144 to Pro152 of the flexible loop Arg141–Glu155 in PAP9 are not observed in the electron density.

Sequence comparisons between PAP9 and SODs of the PDB showed that the flexible C-terminal part (Gly231 to Asp259) of PAP9 is not observed in the sequences of SODs of the PDB. The longest C-terminal extension is observed in FeSOD of *Helicobacter pylori* (PDB entry: 3CEI) (Esposito et al., 2008). However, it is 19 residues shorter than in PAP9 and is folded as a kinked α-helix that interacts with the N-terminal domain. The 29 last residues unobserved in the electron density map of PAP9 are found in several sequences reported as plastid SODs. Indeed, the PAP9 C-terminal part alone, used in alignment searches of the UniProtKB database restricted to plants, matches FeSODs; some of which being not annotated as plastid-localized, despite individual detection of a chloroplast transit peptide using the ChloroP prediction tool. Most of hits are *bona fide* PAP9 orthologous SODs, and the C-terminal sequence represents a signature of this protein family. In addition, the sequence homology between PAP9 and PAP4/FSD3 (MW: 25657.94 Da) from *A. thaliana* is very high, suggesting that both FeSODs have a similar fold. However, PAP4 does not have the C-terminal extension found in PAP9. PAP9 and PAP4 should be functionally distinct and partially redundant as suggested by comparison of individual light-green phenotypes to the more severe albino phenotype of the double mutant (Myouga et al., 2008).

### Solution-State NMR Analyses

**FIGURE 8:**
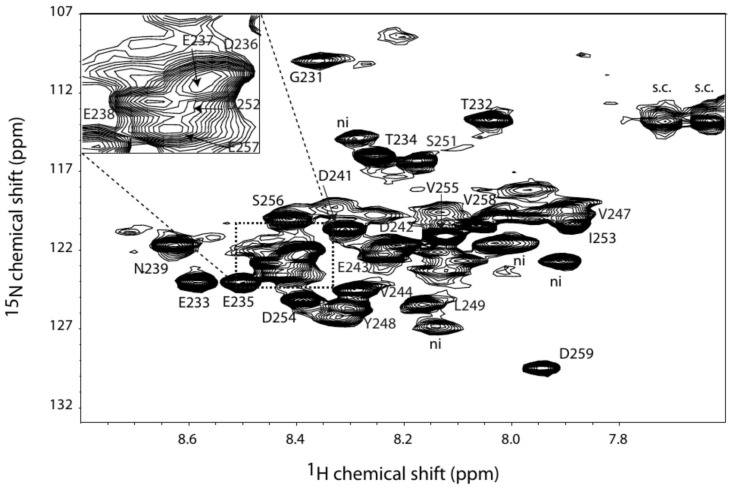
^1^H-^15^N correlation spectrum of PAP9 with the assigned amino acid residue labels annotated “ni” standing for not identified.

Two segments, suggesting a dynamic structure, are not observed in the crystal structure of PAP9, i.e., the loop Arg141–Glu155 and the C-terminal part Gly231–Asp259 and are supposed to behave a fast dynamic. In order to further investigate the structural and dynamic properties of these unseen parts in the PAP9 crystal structure, we produced ^15^N,^13^C-6His-PAP9. In our conditions (see section “Materials and Methods”), only about forty peaks can be observed above the background in the ^15^N-SOFAST spectrum in agreement with the presence of some dynamic residues. The most intense residues have an apparent rotational correlation time of 3 ns measured using [^15^N,^1^H]-TRACT technique (Lee et al., 2006), a value near those expected for free peptides or small proteins such as ubiquitin. In the other hand, the translational diffusion coefficient measured using DOSY experiment at 293 K is of 7 × 10^–7^ cm^2^/s, indicating that PAP9, from the point of view of translational diffusion, behaves like an object of 80 kDa. For such molecular weight, the residues located in the structured regions of the protein are expected to be line broadened supporting the fact that only the flexible residues can be observed in the NMR spectra. These results indicate that the observed residues have a fast movement while being included in a much larger species. We performed a set of 3D-experiments to assign these residues: HNCA, HNCACB, and HNCOCANH. Of these residues, only fifteen present detectable correlations in HNCACB experiments. A first analysis allows characterizing unambiguously a GTxTx sequence that corresponds only to the GTETE sequence located in the C-terminal tail of PAP9. In order to help to identify other residues within this part and characterize secondary structures, we studied a peptide composed of the 34 last residues of PAP9. We have entirely assigned the protons and carbons of the peptide using homonuclear TOCSY, NOESY, and ^13^C-HSQC experiments at natural abundance. SSP program (Marsh et al., 2006) using Cα, Cβ, Hα chemical shift data sets show that the peptide does not present any secondary structure propensity at all (Supplementary Figure 3). In the same way, the ^13^C-HSQC experiment of the integer ^15^N,^13^C-6His-PAP9 presents the very similar correlations than those observed for the peptide (Supplementary Figure 4), strongly suggesting that the C-terminal tail in 6His-PAP9 is also dynamic. Analysis of the observable Cα and Cβ chemical shift values in the protein together with comparison of those of the peptide allowed us to assign the Gly231–Glu238 and the Ser251–Asp259 stretches. Assignments of Asn239, Val247–Leu249 can be proposed on basis of the HNCA experiment. The assigned ^1^H-^15^N correlation spectrum of 6His-PAP9 is shown in Figure 8. No residue of the Gln226-Glu230 stretches were identified in agreement with their position in the last helix of the protein. Interestingly, the correlations of the residues, when observable, located in the middle of the tail: Asn239-Asp250 showed weaker intensities than those in the Gly231–Glu238 and Ser251–Asp259 stretches.

## Discussion

In Angiosperms, the developmental program following germination in the dark is skotomorphogenesis. Inside the cell, chloroplast biogenesis is blocked, allowing for the formation of yellow etioplasts without the chlorophylls. After light perception etiolated seedlings start the photomorphogenesis program leading to chloroplast biogenesis (Liebers et al., 2018). This essential step toward photo-autotrophy involves the rapid assembly of the photosynthetic apparatus within the thylakoid membranes. Jointly, chlorophylls are quickly synthesized from the stored precursors, protochlorophyllides, by the light-activated protochlorophyllide oxidoreductase (POR). Chlorophylls are then inserted in the light harvesting antenna proteins. Transcription of photosynthesis associated plastid genes is ensured by PEP and is rapidly promoted after light perception owing to the PAP assembly into the active PEP complex. Two of the PAPs are FeSODs (Myouga et al., 2008; Steiner et al., 2011). FeSODs catalyze the dismutation of superoxide radicals into peroxides and may protect the transcriptional machinery from the newly acquired photosynthetic capacity (Pfannschmidt et al., 2015). Once the chloroplast is formed and fully photosynthetically active, the PEP activity substantially decreases.

Transmembrane translocation of PAP9 into the chloroplast results from the recognition of its N-terminal plastid transit peptide by the transmembrane TOC/TIC machinery. Fluorescence microscopy experiments showed that PAP9 is mainly located in the chloroplast stroma (Figure 3); the stroma localization may result from the lack of developed thylakoids in onion epidermal cells. Therefore, the predicted nuclear localization sequence observed within the cTP (Figure 2A and Supplementary Table 4) may not serve a localization purpose. It is cleaved off instead during the chloroplast import leading to a mature protein of 30,848 Da as observed using mass spectrometry analysis in denaturing conditions (Figure 4A). The native MS data indicated that PAP9 assembles as dimers. Monomers were also detected, suggesting protein dynamics during assembly. The ionization efficiency of the different oligomeric states affects the relative abundance of the different species in the MS spectra. Therefore, it is not possible to judge whether the monomers are more abundant that the dimers. Moreover, the native MS experiments were performed at 5 μM concentration and in ammonium acetate, which is a different buffer used for purification, NMR, and crystallographic experiments. The buffer conditions may affect the relative abundance of the species.

In the crystals, PAP9 is a symmetric dimer (Figure 5) as revealed by the low RMSD values between both monomers. The buried surface of the dimer interface suggests that the dimer is the biological form of PAP9. The FeSODs and MnSODs are active as dimeric or tetrameric (dimer of dimers) enzymes (Perry et al., 2010). In the PEP, PAP9 has been observed as a monomer (Steiner et al., 2011); a form of the protein also observed in our mass spectrometry analyses. The main difference between PAP9 analyzed here, and the FeSODs or MnSODs, is the metal ion bound to the catalytic site. In our crystal structure a zinc ion, instead of an iron ion, is penta-coordinated by a water molecule, supposed to mimic the position of the hydroxide ion (Muñoz et al., 2005), the His31, His83, Asp182, and His186 side chains (Figure 6) as observed in the cytoplasmic FeSODs and MnSODs. The zinc ion cannot be the catalytic ion to perform the dismutation of superoxide since it has only the redox state II, in opposition to Fe and Mn that both have several redox states from II to VI and II to VIII, respectively. Since PAP9 is an active FeSOD even when overexpressed in *E. coli* (Myouga et al., 2008), the replacement of Fe by Zn occurred during either overexpression or purification of the protein although the metal center is hidden from the solvent by the conserved side chains of His35, Tyr39, Gln79, and Trp184 (Figure 6). As observed in FeSODs and MnSODs, His186 from the catalytic site of one monomer interacts also with the Glu185 carboxylate group from the other monomer. All these observations suggest a conserved catalytic mechanism in cytoplasmic and plastid FeSODs.

The main difference between PAP9 and the other FeSODs, and even MnSODs, is the additional residues of the C-terminal part. In the crystal structures of PAP9-6His and 6His-PAP9, no electron density was observed for the 29 last residues of the C-terminal part resulting from flexibility. Proteolysis can be excluded since the correct molecular weight of the 6His-tagged PAP9 was observed using mass spectrometry (Figure 4A). The flexibility does also not result from the construction of the over-expressed recombinant protein since the electron density of the C-terminal part is not observed for 6His-PAP9. The only observable residues of ^13^C,^15^N-6His-PAP9 using NMR correspond essentially to the C-terminal residues whose dynamic is identical to that of the free peptide (Supplementary Figure 4). This result clearly shows that the C-terminal part is flexible with its central part (weaker intensities of correlations) not as free as the two other parts, probably due to some interactions of this part with residues at the protein surface. As in FeSOD from *V. unguiculata* (Muñoz et al., 2005), no electron density is observed for residues Val144 to Pro152 of the loop Arg141–Glu155 suggesting flexibility in cytosolic FeSODs (FSD1) from plants and PAP9. The C-terminal extension observed in PAP9 could then allow distinguishing between PAP9, as a component of the PEP, and other plant FeSODs. We hypothesize that the C-terminal tail anchors PAP9 to the PEP complex and its observed flexibility arises from the isolation of a subunit that normally belongs to a larger multisubunit complex.

The C-terminal part of the protein had strongly changed during evolution (Figures 1, 2). It is absent in early clades of the green lineage. A first significant C-terminal modification is found in Charales and Physcomitrella while a second longer fragment appears in *Selaginella*. Such events are dating back to the conquest of fresh waters and terrestrial life. It is then possible that the C-terminal part could have appeared along with a complete set of new features for controlling chloroplast transcription; namely the assembly of PEP-PAP complex. The acquisition of these features, including SOD activities in a stoichiometry of four units per complex (three PAP4 and one PAP9), may provide sufficient protection of the organelle while the photosynthetic cells are exposed to a more oxidizing environment. This C-terminal part is totally absent in Gymnosperms, which seem to have evolved a completely different strategy of photo-autotrophy acquisition with, for example, no light regulation of chloroplast biogenesis since seedlings can green in darkness.

The PEP is composed of at least 16 subunits of unknown structures. Interactions between some of them were only reported by using non-direct observations, using yeast-two-hybrid assays (Yu et al., 2013) and fluorescent microscopy (Myouga et al., 2008). We have recently shown by NMR that PAP5 interacts with PAP8 (Liebers et al., 2020). PAP9 was proposed to interact with PAP4 therefore forming a hetero-complex of FeSODs (Myouga et al., 2008), and we show here that PAP9 can have several oligomeric states. Surprisingly, neither this heterocomplex nor the PAP9 dimer have been described (Steiner et al., 2011) suggesting that the PEP is probably a dynamic complex, still poorly characterized at the level of its structure and composition.

## Data Availability Statement

The datasets presented in this study can be found in online repositories. The names of the repository/repositories and accession number(s) can be found below: http://www.wwpdb.org/, 7BJK.

## Author Contributions

RB and DC designed the research. AF, PG, EBE, LS, SSM, RB, and DC performed the research. EBE and LS contributed mass spectrometry data. AF and PG contributed NMR data. RB and DC wrote the manuscript with contributions from AF, PG, EBE, LS, and TP. All authors approved the manuscript.

## Conflict of Interest

The authors declare that the research was conducted in the absence of any commercial or financial relationships that could be construed as a potential conflict of interest.

## Footnotes

1 https://www.ebi.ac.uk/Tools/msa/clustalo/

2 http://www.cbs.dtu.dk/services/ChloroP/

3 http://nls-mapper.iab.keio.ac.jp/cgi-bin/NLS_Mapper_form.cgi

## Supplementary Material

Supplementary Figure 1 Enzymatic assay of PAP9. The superoxide dismutase activity of PAP9 was tested using pyrogallol. The pyrogallol auto-oxidation was followed by monitoring the absorbance increase at 420 nm. After 180 s, PAP9 at several concentrations [50 (orange), 100 (gray), 200 (yellow), 500 μM (light blue), and 1 mM (green)] or 5 μM Mn-SOD (dark blue) were added into the medium and the absorbance was monitored for further 3 min.

Supplementary Figure 2 Transiently expressed PAP9-GFP in onion epidermal cells. N, nucleus; str, stromule; p, plastid. The red arrowhead points to the absence of red fluorescence in stromules.

Supplementary Figure 3 Secondary structure propensity (SSP) scores for the Cter-PAP9 peptide (circles) and the C-terminal tail of integer PAP9 (squares) using ^13^Cα, ^13^Cβ, and Hα chemical shifts. Between residues 245 and 252, the SSP score (star) was obtained from the ^13^Cα and Hα chemical shifts only. Positive values represent α-structure propensity and negative values represent β-structure propensity. The SSP is near zero along the sequence indicating the absence of any secondary structure in the peptide. The numbering of the residues corresponds to the whole protein.

Supplementary Figure 4 Overlay of ^1^H-^13^C correlation spectra (sensitivity-enhanced HSQC) of PAP9 and the Cter-PAP9 peptide. The peptide signals and the PAP9 peaks are shown in red and in black, respectively. All resonances of the peptide are observable in the PAP9 spectrum indicating the presence of the same mobility in the C-terminal tail of PAP9.
