# Supplementary figures and images for: The Plastid-Encoded RNA Polymerase-Associated Protein PAP9 Is a Superoxide Dismutase With Unusual Structural Features
Source: Front Plant Sci. 2021 Jun 30;12:668897. doi: 10.3389/fpls.2021.668897 (PMC8278866; doi:10.3389/fpls.2021.668897)

Figure S0

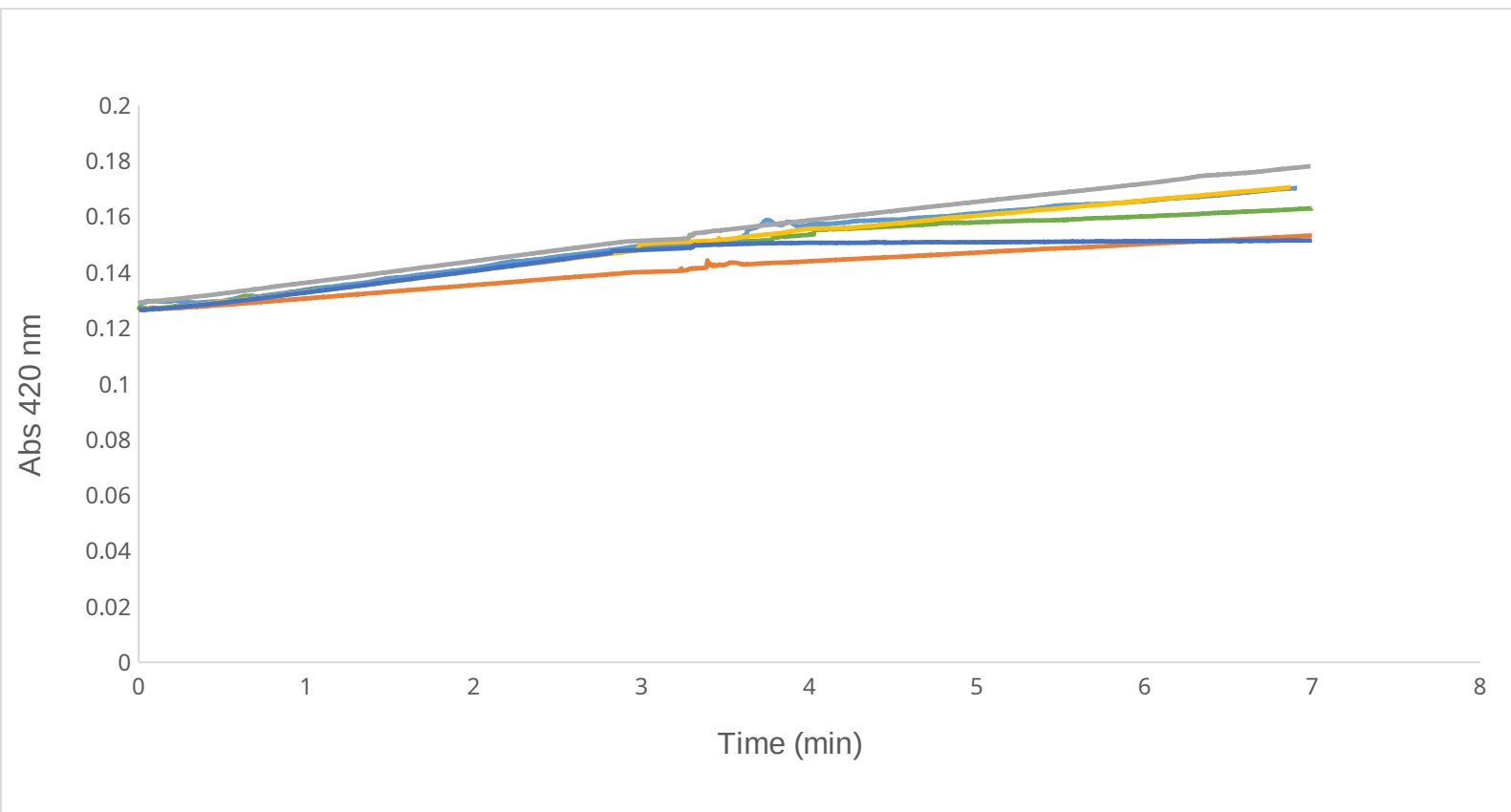

Figure S1

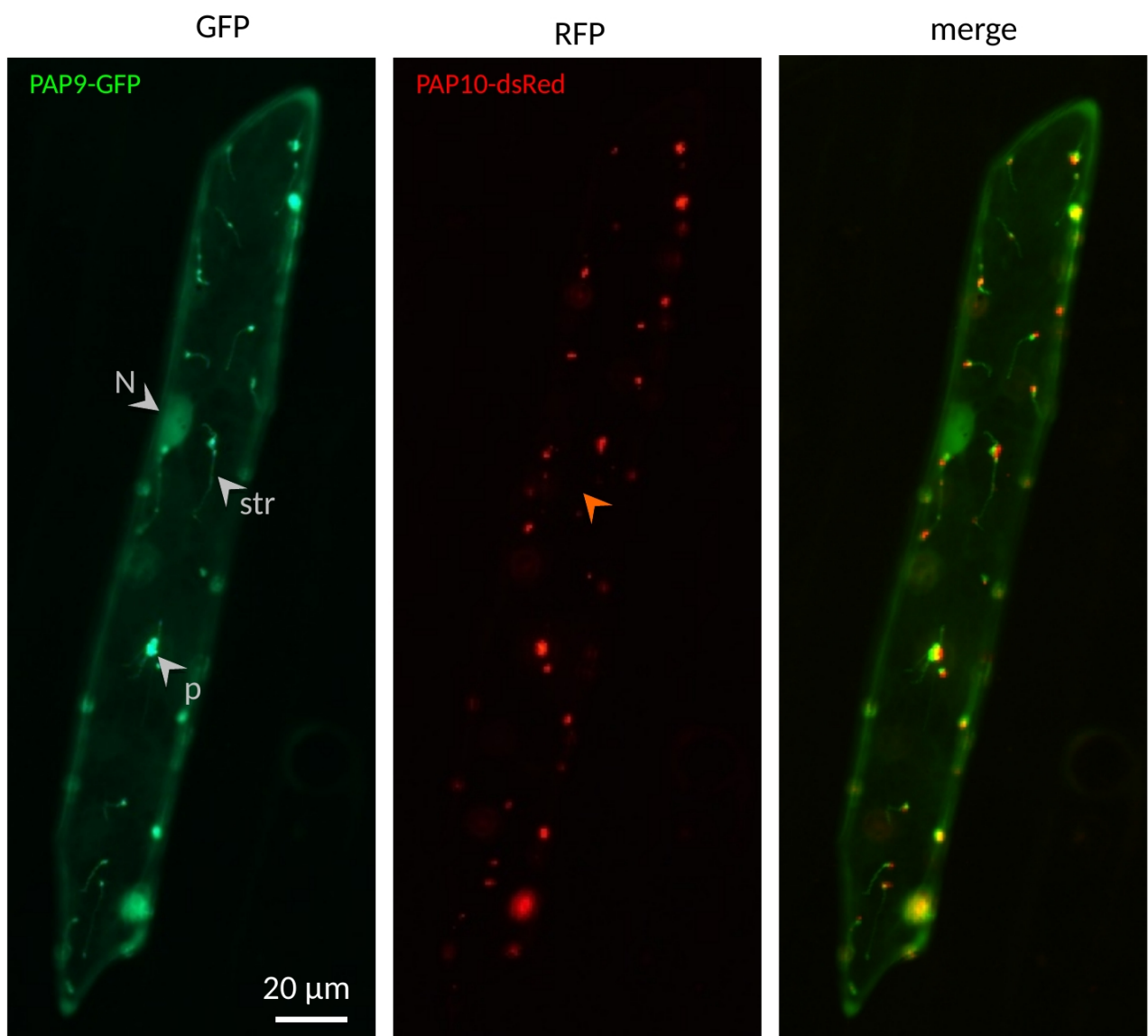

Figure S2

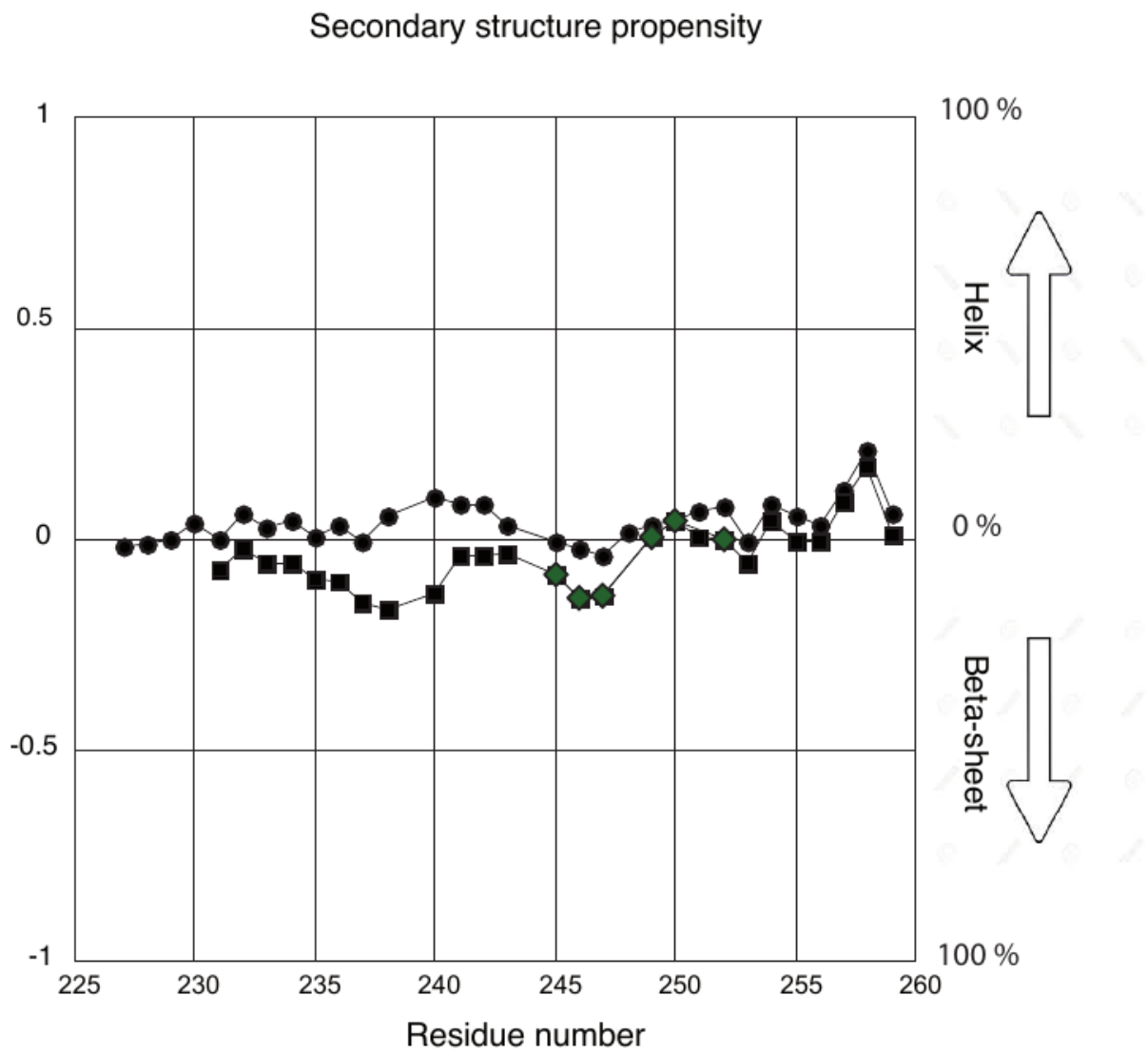

Figure S3

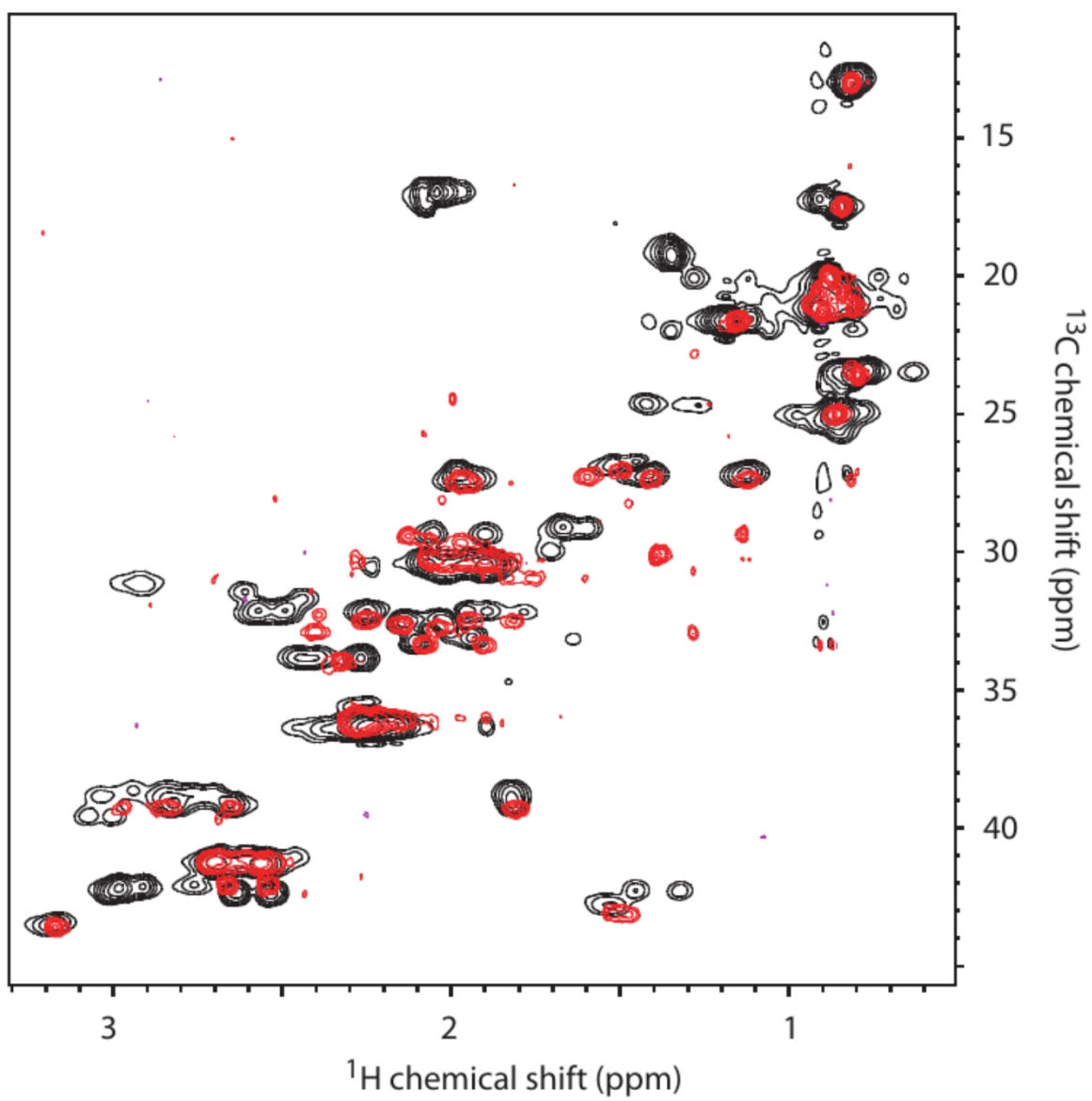

Supplement: Supplementary Figure 1 — Enzymatic assay of PAP9. The superoxide dismutase activity of PAP9 was tested using pyrogallol. The pyrogallol auto-oxidation was followed by monitoring the absorbance increase at 420 nm. After 180 s, PAP9 at several concentrations [50 (orange), 100 (gray), 200 (yellow), 500 μM (light blue), and 1 mM (green)] or 5 μM Mn-SOD (dark blue) were added into the medium and the absorbance was monitored for further 3 min. [file Data_Sheet_1.PDF]
